# Intra‐operative application of ultrasonography (USG) for reduction of zygomatic arch fracture

**DOI:** 10.1002/ccr3.5067

**Published:** 2021-11-11

**Authors:** Pradeep Acharya, Ashok Dongol, Anjani Kumar Yadav, Nilima Bhattarai, Mehul Rajesh Jaisani

**Affiliations:** ^1^ B.P. Koirala Institute of Health Sciences Dharan Nepal; ^2^ Dantakali Dental Hospital and Research Centre Dharan Nepal

**Keywords:** fracture, reduction, ultrasound, zygomatic arch

## Abstract

Zygomatic arch fractures are the most common facial fractures or second in frequency after the nasal fractures. The high incidence of zygomatic fractures probably relates to its prominent position in the facial skeleton; hence, it is frequently exposed to fractures. This case report presents an left‐sided isolated zygomatic arch fracture after subjected to routine investigations and radiographs like submentovertex and CT scans. The patient was operated under general anesthesia for the reduction of zygomatic arch by Gille's temporal approach with the use of Ultrasound intra‐operatively. Recommendation for the use of ultrasonography in the identification of zygomatic arch fractures intra‐operatively operatively.

## Graphical abstract

Ultrasonogram can be considered as the imaging of choice when there is a contraindication to CT or conventional radiograph by reducing the overall amount of radiation. Which also can be the another treatment modalities for the surgical management of close reduction of zygomatic arch fracture.

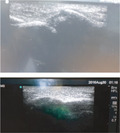

## 1 BACKGROUND

Zygomatic arch, which is the connection between the zygomatic process and the zygomatic bone, forms the most protruding outline of the midface. Depressed zygomatic arch fracture can cause pain and trismus due to hindrance on movement of the coronoid process of mandible. The main purpose of surgical intervention in zygomatic arch fracture is to restore its anatomy and to provide freedom of mandibular movement.^1^ The type, location, magnitude and direction of displacement of zygomatic arch fractures can be determined by plain film radiograph like submentovertex view (SMV), town's view, and CT (computed tomography). Although ultrasound has traditionally been used in orbital and ocular diagnosis, but its role in maxillofacial trauma is less widely recognized. McCann et al^2^ and Friedrich et al^3^ in their separate study have demonstrated the application of ultrasound in visualization of the zygomatic arch fracture and zygomatico‐maxillary complex fracture.

It is safe, inexpensive, non‐invasive, less dependent on patient cooperation, portable so easy to take the image during pre‐, intra‐, and postoperative period, easily reproducible and gives information in real‐time. Thus, this case report has demonstrated the utility of ultrasonography in confirmation of reduction of zygomatic arch fracture both intra‐ and post‐operatively.

## 2 CASE PRESENTATION

**FIGURE 1:**
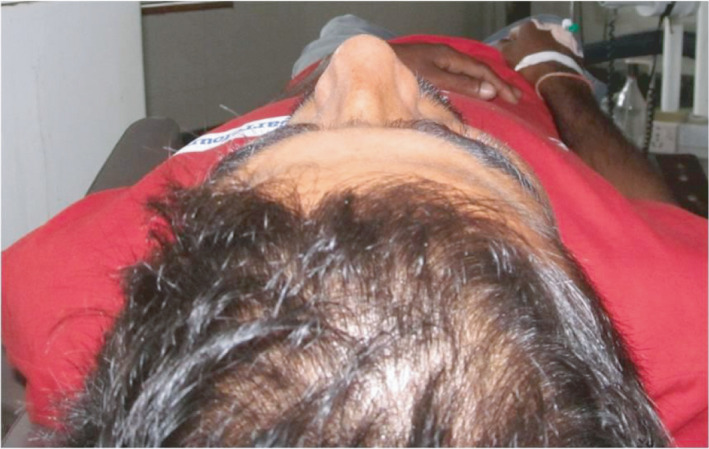
Bird's eye view shows flattening of left zygomatic prominence

**FIGURE 2:**
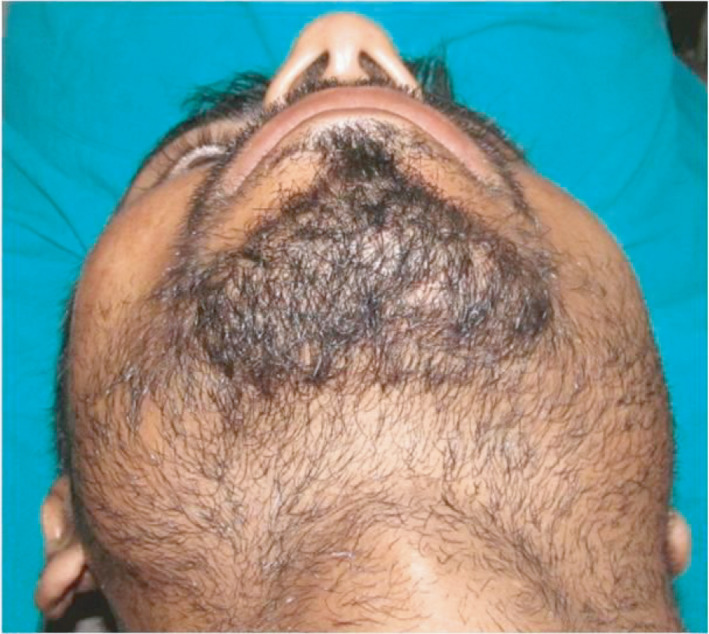
Worm's eye view shows flattening of left zygomatic prominence

**FIGURE 3:**
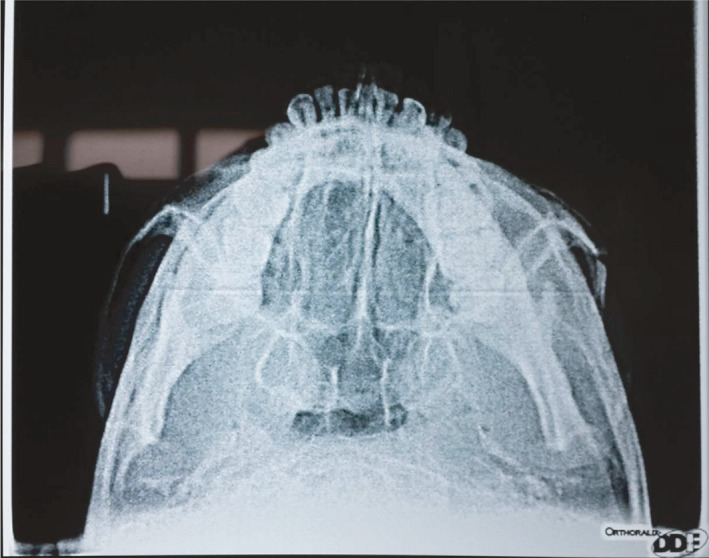
Submento‐vertex view shows left zygomatic arch fracture

**FIGURE 4:**
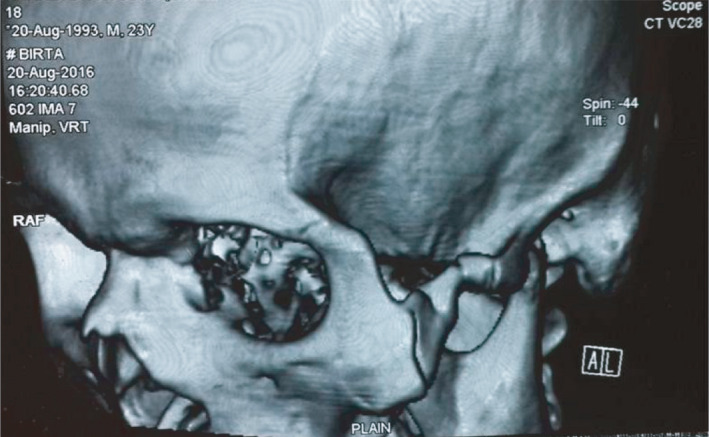
Pre‐operative 3D view of CT scan of same patient

**FIGURE 5:**
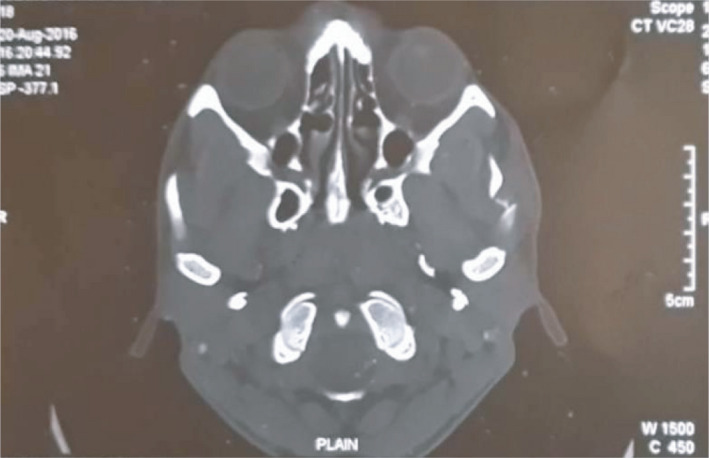
Coronal views of the same patient

A 23‐year‐old male patient reported to the department of oral and maxillofacial surgery with a chief complaint of swelling and pain in left side of face. The patient gave a history of fall from height causing trauma to face 8 days back at the time of presentation. On examination, a depression was found on the left pre‐auricular region (Figures 1 and 2). Palpation over the same area revealed step deformity and tenderness. Patient had restricted mouth opening; however, the occlusion was satisfactory. The patient was subjected to routine investigations and radiographs like submentovertex view and CT scan. Both the submentovertex (Figure 3) and CT scan (Figures 4 and 5) reveal left side zygomatic fracture with depressed arch. A diagnosis of left‐sided isolated zygomatic arch fracture was made based on the clinical examination and radiographs.

**FIGURE 6:**
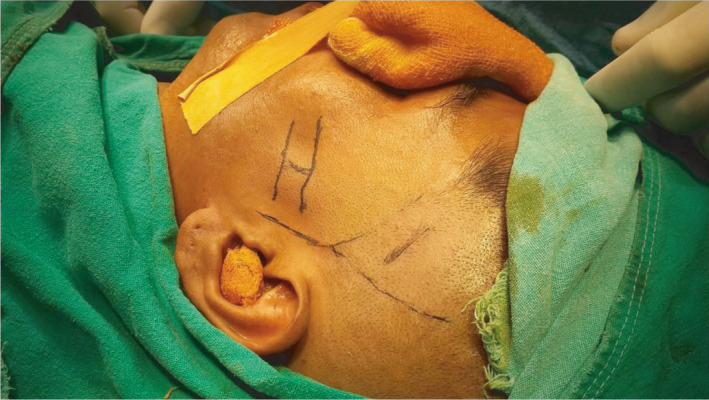
Marking for the location of arch before reduction

**FIGURE 7:**
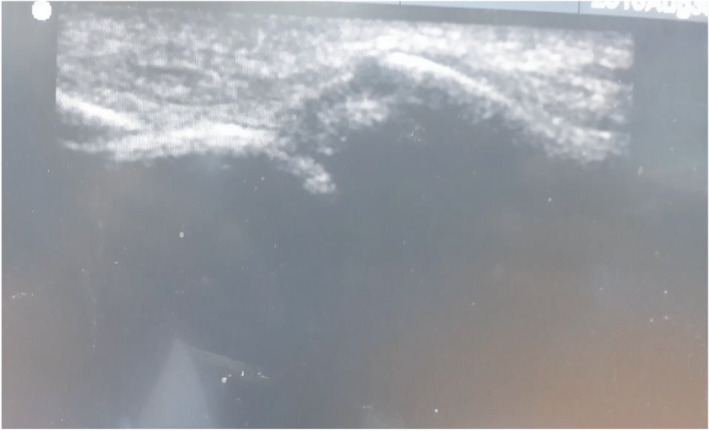
Pre‐op USG of left side of zygomatic arch fracture

**FIGURE 8:**
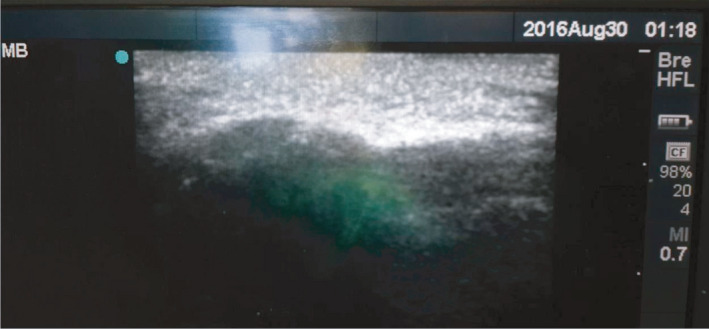
Post‐op USG of left side of zygomatic arch reduced fracture

The case was posted for surgery under general anesthesia. Pre‐operatively USG was used to identify the zygomatic arch fracture. Marking was done (Figure 6). Pre‐op USG shows discontinuation of left side of zygomatic arch due to fracture (Figure 7). Reduction of the zygomatic arch fracture was done by Gille's temporal approach using Rowe's zygomatic elevator. Post‐reduction USG was done to evaluate the fracture site which shows the reduced arch (Figure 8). Post‐operative recovery was uneventful with good mouth opening and with no cosmetic deficit.

## 3 DISCUSSION

Zygomatic arch is the second most common site of facial bone to get fractured and its treatment requires outmost care as it has both cosmetic and functional significance. Conventional plain radiography and computed tomography (CT) scans are the basic diagnostic tools for maxillofacial injuries. Computed tomography (CT) can provide three‐dimensional assessment of fracture, but high radiation exposure, high cost, and difficulty in transporting make its intra‐operative use to assess the reduction difficult.^4^ Further, it cannot be used in pregnant women and in those with cervical spine injuries. Again, fluoroscan assisted closed reduction using C‐arm has the same problem of high exposure to radiation^5^ for the use of intra‐operatively. Although, the use of USG in dentistry has been increasingly developed and widely studied in recent years and its role in maxillofacial trauma surgery is less recognized.^6^ Sonographic evidence may be treated as an alternative diagnostic imaging modality to radiology by which the use of conventional radiographs may not be required.^7^ USG has shown high accuracy in the detection of nasal bone fracture with a sensitivity ranging from 90% to 100%, specificity of 98%–100% and high predictive value^8^ and so it can also be used in assessment of zygomatic arch fracture pre‐ and post‐operatively. In this case report, the patient`s zygomatic arch fracture was observed clearly in USG (Figure  7).

The zygomatic arch fracture is commonly managed by indirect reduction without fixation which is done through various approaches like Gille's temporal approach,^9^ BalaSubrahmaniam upper buccal sulcus approach, Quinn approach,^7^ and Keen's lateral coronoid approach. The patient in the present case report is also managed by close reduction, but with the guidance of ultrasonogram to confirm the accurate reduction. Although gross swelling and emphysema make the ultrasonographic visualization of bony surfaces difficult, this problem was overcome by choosing an ultrasound frequency of 7.5 MHz or less.

Reduction of the zygomatic arch fracture is conventionally done by blind method, and the position of the fragments is usually confirmed by radiography or palpation during the operation. Radiography is not always feasible because of difficulties in managing the patient or the risk of X‐ray exposure, and palpation by the surgeon is often unreliable.^10^ Conversely, ultrasonography is non‐invasive, safe, easily reproducible, and portable and gives information in real‐time, and thus, it overcomes the disadvantages of radiography and palpation.^10^

In this study, the fracture reduction of zygomatic arch was confirmed by ultrasonography intra‐operatively and re‐confirmed by submentovertex view post‐operatively. Use of USG in fracture reduction was followed in this case as it is simple and relatively easy with minimal or no complications and the armamentarium required for this technique is readily available. Thus, ultrasonography can be a reliable method as an alternative to other imaging modalities in cases of close reduction for zygomatic arch fractures.

## 4 CONCLUSION

Considering the better outcome and good reliability of USG in the present case report, we strongly recommended the use USG as an accurate adjunct to conventional radiography in the identification of zygomatic arch fracture intra‐operatively as the overall amount of radiation, cost, expenses, and time are reduced.

## CONFLICT OF INTEREST

All the authors have no conflict of interest to declare.

## AUTHOR CONTRIBUTIONS

Pradeep Acharya prepared main manuscript. Ashok Dongol and Anjani Kumar Yadav involved in the management of patients. Nilima Bhattarai collected photographs and edition. Mehul Rajesh Jaisani guided and reviewed the article before submit for the publication.

## ETHICAL APPROVAL

Consent to participants was taken.

## CONSENT

Taken.

## Data Availability

Not Applicable.

## References

[1] Elizabeth K , Smith SE , Frates MC , Caterson EJ . Use of high frequency ultrasound guidance for intraoperative zygomatic arch fracture reduction. J Cranio‐Max‐facSurg. 2013;24:6. 10.1097/SCS.0b013e3182a21038 24220399

[2] McCann J , Brocklebank M , Ayoub F . Assessment of zygomatico‐orbital complex fractures using ultrasonography. Br J OralMaxillofac Surg. 2000;38:525. 10.1054/bjom.2000.0501 11010787

[3] Friedrich RE , Heiland M , Bartel‐Friedrich S . Potentials of ultrasound in the diagnosis of midfacial fractures. Clin Oral Invest. 2003;7:226‐229. 10.1007/s00784-003-0232-5 14648259

[4] Westendorff C , Gulicher D , Dammann F , Reinert S , Hoffmann J . Computer‐assisted surgical treatment of orbitozygomatic fractures. J Craniofac Surg. 2006;17:837‐842. 17003608 10.1097/01.scs.0000221523.80292.93

[5] Pedemonte C , Saez F , Vargas I , et al. C‐arm as intraoperative control in reduction of isolated zygomatic arch fractures: a randomized clinical trial. Oral Maxillofac Surg. 2016;20:79‐83. 26546376 10.1007/s10006-015-0531-4

[6] Mc Cann PJ , Brockelbank LM , Ayoub AF . Comparision of ultrasonography with submentovertex film and computed tomography scan in the diagnosis of zygomatic arch fracture. British J Oral Maxillofac Surg. 2000;38:525‐529. 10.1054/bjom.2000.0501 11010787

[7] Courtney DJ . Upper buccal sulcus approach to fractures of the zygomatic complex—a retrospective study of 50 cases. Br J Oral Maxillofac Surg. 1999;37(6):464‐466. 10.1054/bjom.1999.0010 10687908

[8] Adeyemo WL , Akadiri OA . Review on applications of ultrasonography in dentomaxillofacial region. Int J Oral Maxillofac Surg. 2011;40:655‐666. 21377837 10.1016/j.ijom.2011.02.001

[9] Ogden GR . The Gille’s method for fractured zygomas—an analysis of 105 cases. J Oral Maxillofac Surg. 1991;49(1):23‐25. 10.1016/0278-2391(91)90261-J 1985179

[10] Akizuki H , Yoshida H , Michi K‐I . Ultrasonic evaluation during reduction of zygomatic arc fracture. J Cranio‐Max‐facSurg. 1990;18:263‐266. 10.1016/s1010-5182(05)80428-7 2212025

